# Refined versus Extra Virgin Olive Oil High-Fat Diet Impact on Intestinal Microbiota of Mice and Its Relation to Different Physiological Variables

**DOI:** 10.3390/microorganisms7020061

**Published:** 2019-02-23

**Authors:** Nieves Martínez, Isabel Prieto, Marina Hidalgo, Ana Belén Segarra, Ana M. Martínez-Rodríguez, Antonio Cobo, Manuel Ramírez, Antonio Gálvez, Magdalena Martínez-Cañamero

**Affiliations:** 1 Área de Microbiología, Departamento de Ciencias de la Salud, Universidad de Jaén, Paraje de Las Lagunillas s/n, Jaén 23072, Spain; mnmc0001@red.ujaen.es (N.M.); mhidalgo@ujaen.es (M.H.); acmolino@ujaen.es (A.C.); agalvez@ujaen.es (A.G.); 2 Área de Fisiología, Departamento de Ciencias de la Salud, Universidad de Jaén, Paraje de Las Lagunillas s/n, Jaén 23072, Spain; iprieto@ujaen.es (I.P.); asegarra@ujaen.es (A.B.S.); msanchez@ujaen.es (M.R.); 3 Departamento de Estadística e Investigación Operativa, Universidad de Jaén, Paraje de Las Lagunillas s/n, Jaén 23072, Spain; ammartin@ujaen.es

**Keywords:** olive oil, polyphenols, butter, next generation sequencing, gut microbiota

## Abstract

Extra virgin olive oil (EVOO) has been reported to have a distinct influence on gut microbiota in comparison to other fats, with its physiological benefits widely studied. However, a large proportion of the population consumes olive oil after a depurative process that not only mellows its taste, but also deprives it of polyphenols and other minority components. In this study, we compare the influence on the intestinal microbiota of a diet high in this refined olive oil (ROO) with other fat-enriched diets. Swiss Webster mice were fed standard or a high-fat diet enriched with EVOO, ROO, or butter (BT). Physiological parameters were also evaluated. At the end of the feeding period, DNA was extracted from feces and the 16S rRNA was pyrosequenced. The group fed ROO behaved differently to the EVOO group in half the families with statistically significant differences among the diets, with higher comparative levels in three families—Desulfovibrionaceae, Spiroplasmataceae, and Helicobacteraceae—correlating with total cholesterol. These results are again indicative of a link between specific diets, certain physiological parameters and the prevalence of some taxa, but also support the possibility that polyphenols and minor components of EVOO are involved in some of the proposed effects of this fat through the modulation of the intestinal microbiota

## 1. Introduction

The negative effects of high-fat diets (HFD) on health, mainly favoring the condition known as metabolic syndrome and the worsening of several cardiovascular variables, have long been known [1]. However, it has been only in the last ten years that the effect of the diet on the prevalent microbial taxa that dwell in the intestines [2], and the possible influence of an intestinal microbial misbalance or dysbiosis on animal physiology [3], have been widely acknowledged. Intestinal microorganisms have an essential role in the host homeostasis and a disturbed colonic microbial ecology can lead to numerous disorders of different kinds, not only digestive but also metabolic or even cognitive through the gut–brain axis. Therefore, gut microbiota has emerged as a new and important factor to be considered in the negative consequences of HFD [4]. 

Considering that intestinal microbiota is composed of a large number of bacterial cells, distributed in a high number of taxa with complex ecological relationships among them and with an increasingly more evident metabolic continuous communication with the host, it makes sense to think that changes in the microbiota might have a concomitant effect on the organism [5]. If the large intestine is viewed as a fermenter or a bioreactor, it also seems reasonable to consider that food and the type of diet may be the main or one of the main factors exerting an effect on the microbiota that thrives within. Therefore, when this diet presents certain physiological distinctions, it is not illogical to consider that these variations could be produced, at least in part, throughout the bacterial metabolism, being this step necessary for the physiological outcome. This assumption is especially rational when correlations between the biological traits and the bacterial presence are statistically significant and, hence, a link is proven to happen. However, correlation is not causation and the two variables can change concomitantly for different reasons. One variable can certainly affect the other or vice versa or it could just happen that both of them are equally affected by the specific diet, with no interaction at all between the microbial taxon and the physiological variable. 

For years, nutritionists and researchers have claimed that the impact of lipids is dissimilar depending on their degree of saturation [6,7] and, hence, this different effect has concomitant outcomes as well on the microbiota profile [8]. In particular, monounsaturated fatty acids are considered to be particularly healthy [9], and among them, olive oil has been widely studied because of its prevalent role in the Mediterranean Diet and its appreciated sensorial characteristics [10]. The specific effect of olive oil on intestinal microbiota was consequently highly interesting and the study was undertaken by our laboratory using, first, denaturing gradient gel electrophoresis [11] and, recently, using a 16S ribosomal DNA metagenomic approach [12] as explained further below in this section. 

Virgin olive oil is also enriched in some minority components, such as polyphenols, which are well known for their antimicrobial activity on certain bacteria [13] as well as for their anti-inflammatory and antioxidant effects [14]. Because of this behavior, the specific influence of polyphenols on the intestinal microbiota has similarly been studied in several foodstuffs [15,16]. However, not all commercial olive oils have the intact polyphenol content. Virgin olive oil is extracted mechanically from olives with no chemical intervention and contains relatively high amounts of phenolic compounds and tocopherols. In contrast, if the acidity of the virgin oil is too high; it may undergo a chemical treatment to improve this condition and it is then transformed in refined olive oil (ROO). ROO has the same fatty acid composition as the virgin oil from which it is derived but it looses most of the minority components in the process as well as the palatability and the bouquet [17]. This is why it is commercialized only after 10–15% of virgin olive oil has been added, which improves its organoleptic properties. In fact, virgin olive oil has an average polyphenol content of 150–400 mg/kg while refined olive oil has only a residual presence (0–5 mg/kg) [18]. The loss of the unsaponifiable matter is important since it could change some of the healthy properties attributed to virgin olive oil. This importance is magnified by the fact that the commercialization of ROO is significant since, according to the local Ministry of Agriculture [19], there was 60% consumption of this fat versus 40% consumption of virgin and extra virgin olive oils during 2016 in Spain, the main olive oil producer and one of the leading consumers of this fat in the World. Moreover, ROO is preferred by American consumers over some virgin olive oils, probably because of its milder flavor, according to a recent inquiry [20]. 

In spite of all these facts, few scientific studies have focused on the influence of ROO on health [17,21] and none of them have studied its effect on the intestinal microbiota. Aware of this, we included commercial ROO in the above mentioned analysis [11]. In that report ROO showed different behavior to EVOO and a distinctive microbial profile using denaturing gradient gel electrophoresis. However, microbial taxa were not thoroughly studied, neither was the physiological effect on the host. Recently, using a 16S ribosomal DNA metagenomic approach we compared a standard diet (SD) and two high-fat diets enriched in butter (BT) and extra virgin olive oil (EVOO), respectively [12]. The results obtained showed clear differences both in the fecal microbiota profile and in several physiological variables related to metabolic syndrome. Some of these variables held statistically significant correlations with the percentages of several taxa that increased in the butter-enriched diet but not in the one with EVOO. As ROO was not included then, in the present study we have extended the previous report by incorporating a diet enriched in commercial ROO in order to elucidate differences in microbial taxa between this and the other diets and to look for possible correlations with physiological variables in the host. The results obtained can not only add new data to the influence of ROO on health but also on the specific role of polyphenols on the effect exerted by virgin olive oil.

## 2. Materials and Methods

### 2.1. Animals

Experimental procedures were followed as already described for three of the four diets in reference [12] (SD, standard chow diet; high-fat diets: EVOO, standard chow enriched in virgin olive oil, and BT, standard chow enriched in butter, until reaching 35% of total energy in both cases). In this work, a fourth experimental group fed with ROO diet was also added, consisting of standard chow enriched in refined olive oil until reaching 35% of total energy. As in Prieto et al. [12], standard Panlab A04 chow was used but this time it was supplemented with 20% refined olive oil instead of virgin olive oil or butter. Table 1 shows the composition of the four diets. Briefly, we fed ad libitum 8 male Swiss Webster mice with SD diet, and 9 mice with EVOO, BT, and ROO diets, respectively, making a total of 35 mice, during a 12-week period.

EVOO and butter were obtained and characterized as indicated [12]. ROO was obtained from a large commercial store (Hacendado, Mercadona, Jaén; 76.6% MUFA, 7.1% PUFA, 16.3% SFA). All experimental procedures were reviewed and approved by the Bioethics Committee of the University of Jaén in accordance with 86/609/EEC, initially on 29 December 2010 for project AGR 6340 and extended for project PP2015/08/09. The procedure was followed as described previously [12]. Mice were housed at constant temperature (23 °C), constant humidity (50%), and with a constant day length (12 h) and, twenty-four hours before sacrifice, they were individually placed in metabolic cages so that food intake, water intake, diuresis, body weight (BW), and systolic blood pressure (SBP) could be determined for each animal. After twelve weeks, feces from each mouse were also saved individually right after deposition and total DNA was extracted immediately as indicated below or kept at −80 °C until use. SBP was monitored as previously described [12,22]. At the end of the twelve-week period and after collecting all data (food and water intake, diuresis, body weight and SBP) and feces, animals were anesthetized, and blood samples were obtained through the left cardiac ventricle. Finally they were sacrificed by perfusing them through the same ventricle with saline solution [12]. Insulin, fasting glucose, triglycerides, total cholesterol, and HDL were measured as previously reported [12,22,23]. Leptin and ghrelin concentrations in plasma were also determined as previously reported [12,24].

### 2.2. Bacterial Biodiversity

Fecal bacterial community was studied by pyrosequencing the amplified metagenomic 16S rRNA as already described for three of the diets [12]. Nucleic acids were purified using QIAamp© DNA Stool Kit (QIAGEN, Hilden, Germany and sequencing was performed at Lifesequencing (Valencia, Spain) as described [12] after thirty-five libraries were constructed. Quality control (Q20 threshold) and check for quimeras (UCHIME v. 4.2.40 program, Tiburon, CA, USA) have also been detailed before [12]. Taxonomic levels were assigned through the Ribosomal Database Project Classifier.

### 2.3. Statistical Studies

For the statistical analysis, we followed the procedures described previously [12]. Statistical significant differences in the distributions of the variables of interest according to the type of diet were tested at a 5% of signification by ANOVA or Kruskal–Wallis test depending on whether the assumptions were met or not. When the null hypothesis was rejected, pairwise comparisons were performed by Dunn test with *p*-values adjusted by Bonferroni correction. In addition, for each physiological variable under consideration, multiple lineal regression models were developed using as independent variables those that showed significant differences in ANOVA or Kruskal–Wallis test. The regression models were fitted by stepwise regression and backward elimination. The statistical software used was SPSS 19 IBM (Armonk, NY, USA), R 3.4.4 (Auckland, New Zealand, and Gretl 2018c (San Diego, CA, USA).

## 3. Results

### 3.1. Physiological Parameters

At the end of the experimental period, different physiological variables were measured. Raw data corresponding to BT, EVOO, and SD were previously reported in Prieto et al. (2018) [12]. In the present work, we found that BT-enriched diet produced the highest statistically significant body weight (*p* < 0.05) and SBP levels (*p* < 0.01) of all four diets, as well as the highest plasma insulin levels, which were also significant versus EVOO- and ROO-enriched diet values (*p* < 0.05) (Table 2). Mice fed SD had the lowest statistically significant total cholesterol levels in plasma (*p* < 0.01) and EVOO fed mice had the second lowest levels, being significant versus the values found in the ROO group (*p* < 0.01). It is worth mentioning that there is no cholesterol present either in EVOO or ROO in opposition to BT, where it can be found at about 215 mg/100 g. EVOO also triggered the highest HDL/LDL ratio, being significant versus SD values (*p* < 0.05) as already reported [12], ROO produced the second lowest values but with no statistical significance (Table 2). No significant differences were found in food or water intake, diuresis, and plasma leptin, ghrelin or triglycerides.

### 3.2. Sequencing, Taxa Adscription, Percentage Comparison, and Correlations

A total amount of 264.97 MB from the 35 fecal samples were sequenced, once reads were finally stabilized. Sequences were trimmed and filtered, leaving a final sum of 393958 (540–555 nt of mean length). After performing the blast search, reads were grouped in taxa according to different taxonomical levels, with a final output of 10 phyla, 82 families, 223 genera, and 513 species.

**Figure 1:**
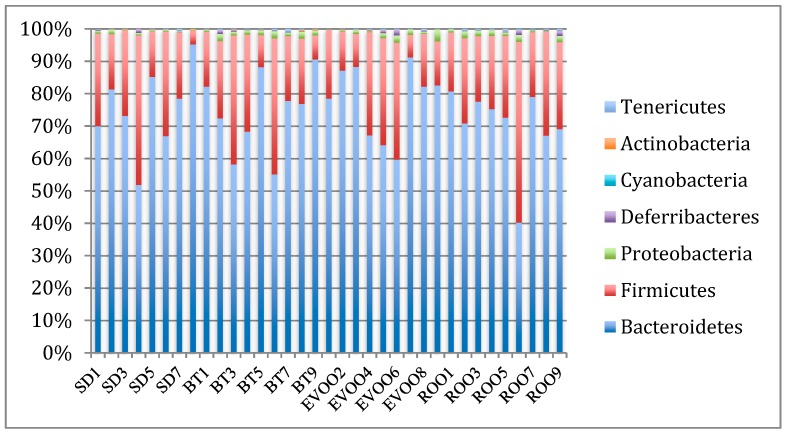
Bacterial distribution, in sequence percentage, at the level of phylum. Each column represents the bacterial fecal community from one mouse, grouped by diet. BT: butter diet; EVOO: extra virgin olive oil diet; ROO: refined olive oil diet; SD: standard chow. SD, EVOO, and BT diet values from Prieto et al. [12].

**Figure 2:**
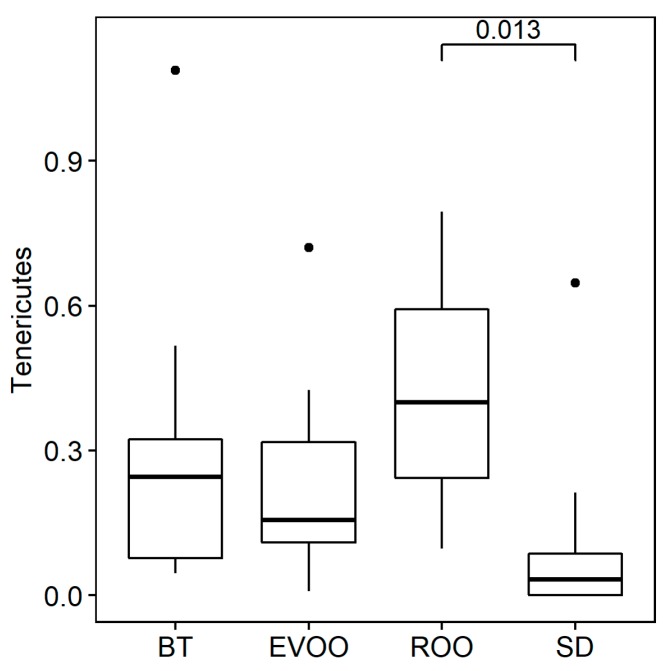
Box plots of presence of the phylum Tenericutes, in percentage of sequences, after feces DNA extraction from mice fed with the different diets. BT: butter diet; EVOO: extra virgin olive oil diet; ROO: refined olive oil diet; SD: standard chow. SD, EVOO, and BT diet values from Prieto et al. [12].

For clarity, Figure 1 shows only the seven most representative phyla. These are complemented with the phyla Synergistetes, Verrucomicrobia, and Deinococcus-Thermus that are present in a very low proportion in one single mouse under SD in the first case and under the ROO diet in the other two. After analyzing the distributions using Kruskal–Wallis test, only the phylum Tenericutes showed statistically significant differences (*p* = 0.023), with a pairwise comparison rending adjusted signification only between the standard and ROO groups (*p* = 0.013; Figure 2). When a regression fit was performed, Tenericutes showed positive correlation with total cholesterol (*R*^2^ = 0.12; *p* = 0.041) and diuresis (*R*^2^ = 0.40; *p* = 0.030).

**Figure 3:**
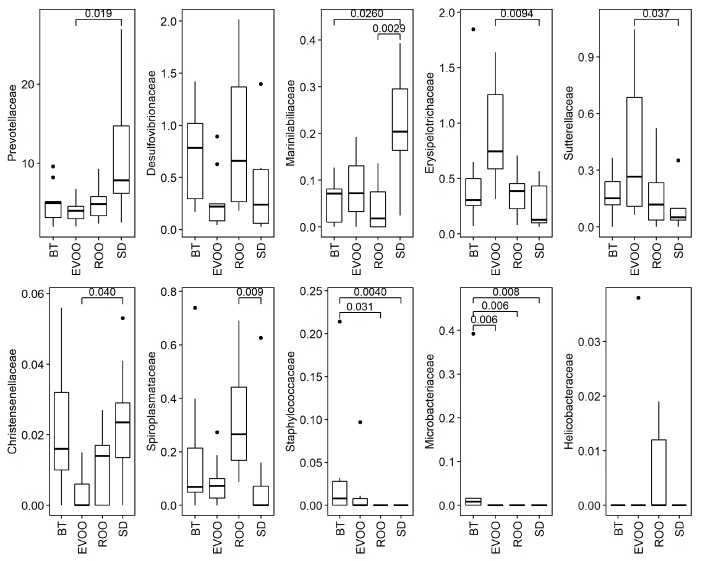
Box plots of presence of the ten families with significant differences (*p* < 0.05), in percentage of sequences, after feces DNA extraction from mice fed with the different diets. BT: butter diet; EVOO: extra virgin olive oil diet; ROO: refined olive oil diet; SD: standard chow. SD, EVOO and BT diet values from [12]. Significant pairwise comparisons adjusted *p*-values are also shown.

At the family level, ten taxa had statistically significant differences among the four groups after a Kruskal–Wallis analysis. Figure 3 shows the box plot diagrams of these families with the corresponding pairwise adjusted *p*-value in the significant cases. As it is shown, EVOO had statistical significant differences with SD in the percentage of Prevotellaceae, Erysipelotrichaceae, Sutterellaceae, and Christensenellaceae, while ROO had differences with ROO in Marinilabiliaceae and Spiroplasmataceae, and with BT in Staphylococcaceae. Additionally, the levels of Microbacteriaceae were significantly different between BT and each one of the three other diets. 

Different multiple linear regression models were fitted to explain each physiological variable, using as independent variables all the families with significant differences. Table 3 shows the results obtained. Only nine families are shown since Christensenellaceae did not retrieve any significant model. 

**Figure 4:**
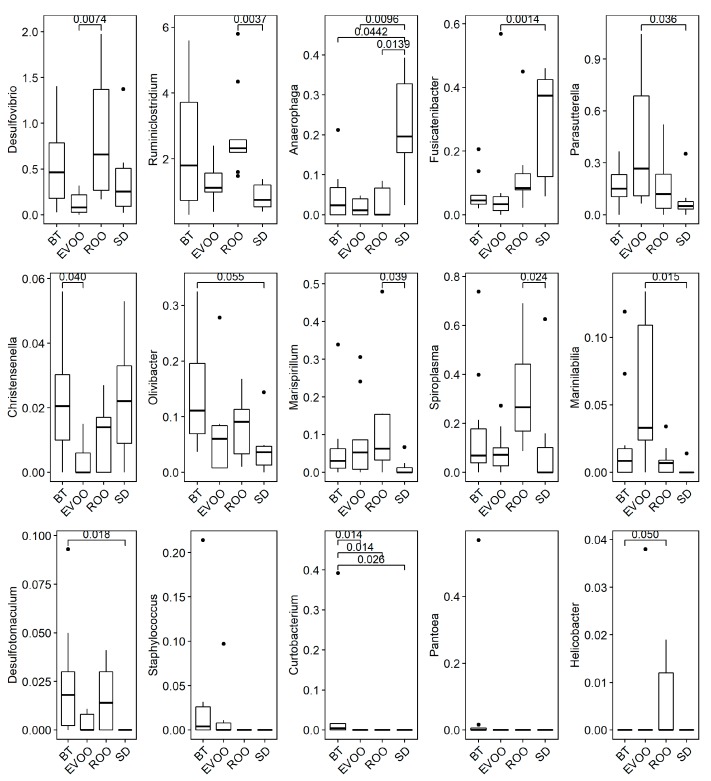
Box plots of presence of the fifteen genera with significant differences (*p* < 0.05), in percentage of sequences, after feces DNA extraction from mice fed with the different diets. BT: butter diet; EVOO: extra virgin olive oil diet; ROO: refined olive oil diet; SD: standard chow. SD, EVOO and BT diet values from [12]. Significant pairwise comparisons adjusted *p*-values are also shown.

With respect to genera, a Kruskal–Wallis analysis indicated that fifteen of the 223 genera detected had statistically significant differences among the diets, as shown in Figure 4, where the corresponding pairwise adjusted *p*-value is also indicated in the significant cases. As specified, EVOO had statistical significant differences specifically with ROO in the percentage of Desulfovibrio; with SD in the percentage of Fusicatenibacter, Parasutterella, and Marinilabilia, and with BT in Christensenella. On the other hand, ROO had statistical significant differences with SD in Ruminiclostridium, Marispirillum, and Spiroplasma, and with BT in Helicobacter. Additionally, the three high-fat diets presented significant differences with SD in Anaerophaga, and BT had significant differences with the other three diets in Curtobacterium. 

Again, for each physiological variable a multiple linear regression was fitted considering as covariates the genera that show significant differences. A high colineation was found between the genera Curtobacterium and Pantoea and, therefore, the analysis was performed twice, each time with one of the two of them. The results, shown in Table 4, were exactly the same in both cases.

## 4. Discussion

The present results demonstrate that there are differences in the intestinal microbiota of mice fed different high-fat diets, including a diet enriched in refined olive oil, and show that these changes correlate with certain physiological variables. 

Olive oil, the main fat in the Mediterranean diet, has long been considered to be healthy and its influence on the intestinal microbiota has recently been studied [11,12,25]. Moreover, correlations between the percentage of specific bacterial taxa and physiological variables related to the metabolic syndrome [12,26] have been reported. Considering the dissimilar composition of the unsaponifiable fraction of different olive oils, a comparison between virgin and refined olive oil can help us to understand the responsibility of polyphenols and other minority components on the beneficial effects of olive oil and the importance of intestinal microbiota on them.

In this work, we analyzed data obtained from mice fed a diet enriched in ROO in comparison with mice fed a diet enriched with EVOO or with BT or a standard chow diet. Differences in microbial percentages as well as in the host physiology and their correlations have been evaluated. After twelve weeks of diets, ten bacterial families showed significant statistical differences upon a Kruskal–Wallis test (Figure 3). In two of them (Prevotellaceae and Marinillabiliaceae) there was a marked distinction between the standard diet and the three high-fat diets. In the case of Prevotellaceae, there was adjusted signification between the SD and EVOO groups (SD versus the other two fats nonadjusted signification was also <0.05; Table S1). This family is related to plant-rich diets with high intake of carbohydrates, fiber and vegetables [27] which fits with the high levels detected in SD, a grain-based chow, and with its significant inverse correlation with total plasmatic cholesterol, not present in this diet. Contrary to this, in another five families—Desulfovibrionaceae, Erysipelotrichaceae, Sutterellaceae, Spiroplasmataceae, and Helicobacteraceae—there were differences among the three high-fat diets. One of the most interesting ones was Desulfovibrionaceae where global comparisons are significant even though adjusted pairwise signification was not detected, but where EVOO-ROO nonadjusted signification showed a *p*-value below 0.05 (Table S1). As we have previously discussed [12] Desulfovibrionaceae are sulfate-reducing bacteria that could be sustained by butter sulfate sources, since chondroitin sulfate, a common dietary supplement of animal origin, has been shown to stimulate Desulfovibrio intestinal growth [28] and, in some instances, they have been related to high-fat diets derived from milk [12,29]. According to our own report [12], this could explain the high presence of this bacterial taxon in the BT fed group in opposition to the EVOO group, also a high-fat diet. However, the unexpected high levels found in ROO in the present study delimitates more this scenario. With no sulfates from animal origin, this family was expected to behave in ROO as it does in the EVOO diet, unless there was an alternative available source of sulfur compounds. Indeed, the extra virgin olive oil used in this work is from organic farming, with no additives pre- or postharvest, and therefore no external components, like sulfur compounds, are present. On the contrary, commercial refined olive oils are mixed stocks from different origins and different cultivation conditions and the presence of other compounds cannot be ruled out. In addition to this, the effect of polyphenols in the low Desulfovibrionaceae levels detected in EVOO cannot be discarded either. Another interesting result is related to the physiological correlations since Desulfovibrio presents a positive correlation with food intake, water intake, diuresis, and total cholesterol (Table 4). 

Spiroplasmataceae is another remarkable case (Figure 3), moreover because it belongs to phylum Tenericutes (Figure 2), also significantly incremented in the ROO group. This family shows significant pairwise differences between ROO and SD and nonadjusted signification between ROO and the other two high-fat diets (with EVOO *p* < 0.05 and with BT *p* < 0.1; Table S1). At genus level, several genera belonging to this family have been detected but only one, Spiroplasma, reaches significance (Figure 4). Spiroplasmataceae correlates highly with total cholesterol (Table 4), which makes sense because these bacteria require cholesterol for growth since they have it in their cell membranes but cannot synthesize it [30,31]. They also require different fatty acids: mainly palmitic acid but oleic acid can also promote growth [30]. The low levels detected in the EVOO group can be related to the statistically significant different levels of total cholesterol present in the two groups, but polyphenol content can also play an important role, since hydroxytyrosol and polyphenols have been described as important antimicrobials for Tenericutes [32,33]. In this case it seems that the increment in bacterial taxa does not produce the physiological change but it is the physiological change that produces the bacterial increment. However, nondetected additional effects of this bacterial increment cannot be discarded and therefore it is important to be aware of this trend. 

There is another family with unexpected significant increments in the ROO group, Helicobacteraceae (Figure 3), conformed by a single genus also significant in this study, Helicobacter (Figure 4), and by a single species, most similar to *H. mastomyrinus*, a microaerophilic, upper gastrointestinal tract, enterohepatic species [34] as most of the species of this family are. Both family and genus are inversely correlated with leptin (Table 3 and Table 4), and it is another result worth taking into consideration, where polyphenols seem to have again an important protective role.

The other two families with different distribution between the ROO and EVOO groups are Erysipelotrichaceae and Sutterellaceae where, in opposition to the other cases, the EVOO group shows the highest values (Figure 3). The first family correlates inversely with insulin and the second one, inversely with leptin (Table 3), although the main significant genus detected in this family also correlates inversely with insulin (Table 4). The link of the EVOO group with bacterial percentages correlating with low levels of insulin was a recurrent result also in our previous report [12] and, therefore, polyphenols are good candidates to play a role in this outcome. 

Finally, two families are present in very low but significantly higher proportions in the BT group than the others: Staphylococcaceae and Microbacteriaceae (Figure 3). These two families are skin dwellers, able to easily survive in the environment and they are conformed in this study by several genera and species, and therefore we do not discard a mouse skin origin favored by the BT-enriched diet. 

In addition to the above genera, there are six additional ones that present significant differences but whose superior taxa do not (Figure 4). These are three genera belonging to the order Clostridiales (Ruminoclostridium, Fusicatenibacter, and Desulfotomaculum), two Proteobacteria (Marispirillum and Pantoea), and one Bacteroidales (Olivibacter), none of them with adjusted significant differences between EVOO and ROO. It is worth noting the case of Desulfotomaculum—another sulfate-reducing bacteria that has a higher presence in BT and ROO—as Desulfovibrio does, although with a slight decrease in ROO with respect to BT that coincides with the loss of correlation with total cholesterol. With respect to the Proteobacteria genera, the five detected belong to the five described proteobacterial lineages (α, β, γ, δ, ε-Proteobacteria) and all of them are present in a higher proportion in one of the high-fat diets. This reinforces the hypothesis that relates this bacterial taxon with fats [12,35], and with LPS-induced metabolic endotoxemia [36]. However, it should be noted that in our present results and in the previous ones [12], EVOO-fed mice present higher percentages only in a β-Proteobacteria, Parasutterella (fam. Sutterellaceae), which does not show any of these outcomes, correlating with lower levels of leptin, insulin, and HDL/LDL. 

As indicated previously, correlation is not causation and the fact of uncovering two variables that evolve in parallel does not automatically indicate that one is the cause of the other. In the case of the present report, a number of physiological variables have proven to modify their values in relation to the presence of certain bacterial taxa but more work has to be done, mainly in controlled gnotobiotic mice models, to undoubtedly show the extend of the responsibility of the microbiota in those effects. The results here presented delimitate the scenario for these future experiments.

## 5. Conclusions

As a summary, we have detected five bacterial families with different percentages between virgin and refined olive oil fed mice. In three of these families (Desulfovibrionaceae, Spiroplasmataceae, and Helicobacteraceae) extra virgin olive oil polyphenols and/or other minority components could contribute to prevent undesirable bacteria from reaching the levels obtained in ROO diet. In another two families (Erysipelotrichaceae and Sutterellaceae), polyphenols may act indirectly to promote these families increment with outcomes that still remain to be completely elucidated. Another prevalent bacterial family, Prevotellaceae, does not seem to be affected by the unsaponifiable fraction, at least in an apparent manner, showing the same behavior in the three high-fat diets, independently of their composition. Finally, the detected specific increment of Spiroplasma and Helicobacter species in the ROO enriched diet should be further studied.

## Figures and Tables

**Table 1:** Nutrient composition and energy content of standard (SD) and high-fat diets enriched with extra virgin olive oil (EVOO), refined olive oil (ROO) and butter (BT).

Diet.	SD		EVOO		ROO		BT	
Composition	g/100 g	% energy	g/100 g	% energy	g/100 g	% energy	g/100 g	% energy
Protein	16.5	20	16.5	14	16.5	14	16.5	14
Carbohydrates	60	72	55	48	55	48	55	48
Fat	3	8	20	35	20	35	20	35
Total Energy (kJ/g)	14.2		19.6		19.6		19.6	

**Table 2:** Metabolic and physiological values in mice at the end of the experiment.

Diet	SD	EVOO	BT	ROO	p
Food Intake (g/day)	3.70 ± 0.65	3.74 ± 0.39	2.76 ± 0.38	3.83 ± 0.41	n.s.
Water intake (mL/day)	8.71 ± 1.61	11.28 ± 1.60	6.67 ± 1.95	7.14 ± 1.10	n.s.
Diuresis (mL/day)	2.44 ± 0.70	2.68 ± 0.65	1.65 ± 0.63	2.41 ± 0.50	n.s.
Body Weight (g)	39.09 ± 1.17	38.62 ± 0.71	42.15 ± 0.61	38.09 ± 0.86	A *
SBP (mmHg)	161.71 ± 11.83	148.11 ± 5.94	190.50 ± 8.53	156.14 ± 19.54	A **
Plasma Leptin (pg/mL)	1929.63 ± 437.86	949.89 ± 230.36	1433.23 ± 226.95	897.33 ± 259.13	n.s.
Plasma Ghrelin (pg/mL)	55.0 ± 17.56	94.3 ± 62.36	78.02 ± 42.82	89.33 ± 14.97	n.s.
Plasma Insulin (mg/100 mL)	1253.28 ± 201.32	685.71 ± 139.57	1518.44 ± 329.97	875.97 ± 132.60	B *
Plasma Glucose (mg/100 mL)	194.13 ± 17.31	175.33 ± 18.95	192.0 ± 17.2	259.63 ± 20.50	C *
Plasma Triglycerides (mg/100 mL)	39.11 ± 11.88	26.01 ± 3.77	48.94 ± 4.89	47.44 ± 8.89	n.s.
Plasma Total Cholesterol (mg/100mL)	49.28 ± 10.53	84.57 ± 9.98	98.67 ± 9.98	115.4 ± 11.39	D **
Plasma HDL/LDL Ratio	0.20 ± 0.02	0.38 ± 0.08	0.32 ± 0.03	0.28 ± 0.02	E *

Given values are mean ± SEM. Standard, EVOO, and BT diet values from [12]. n.s.: not significant; A: differences in BT vs. standard, ROO, and EVOO diets; B: differences in BT vs. EVOO, and ROO diets; C: differences in ROO vs. BT, standard, and EVOO diets; D: differences in standard vs. EVOO, ROO, and BT diets, and EVOO vs. ROO diet; E: differences in standard vs. EVOO diet; * *p* < 0.05; ** *p* < 0.01.

**Table 3:** Regression analysis for the physiological variables studied and those families with statistical differences in total percentage.

Variable	Diuresis (0.23/0.0086)	Leptin (0.79/0.0000)	Insulin * (0.12/0.0456)	Total Cholesterol (0.23/0.0185)	Triglycerides (0.62/0.0000)
Prevotellaceae	n.s.	n.s.	n.s.	−2.73 ± 1.18 (0.0276)	n.s.
Desulfovibrionaceae	n.s.	−309.07 ± 78.84 (0.0006)	n.s.	n.s.	n.s.
Marinilabiliaceae	n.s.	−2112.02 ± 1004.75 (0.0462)	n.s.	n.s.	n.s.
Erysipelotrichaceae	1.55 ± 0.54 (0.0086)	n.s.	−0.461 ± 0.221 (0.0456)	n.s.	n.s.
Sutterellaceae	n.s.	−1026.14 ± 189.08 (0.0000)	n.s.	n.s.	n.s.
Spiroplasmataceae	n.s.	−5033.70 ± 2006.53 (0.0000)	n.s.	60.63 ± 28.27 (0.0399)	n.s.
Staphylococcaceae	n.s.	−5033.70 ± 2006.53 (0.0193)	n.s.	n.s.	110.39 ± 19.70 (0.0000)
Microbacteriaceae	n.s.	n.s.	n.s.	n.s.	22.73 ± 8.61 (0.0130)
Helicobacteraceae	n.s.	−40363.4 ± 8870.42 (0.0001)	n.s.	n.s.	n.s.

For each case, regression coefficient estimate, s.e. and *p*-values are shown. *R*^2^ and *p*-values of the model are also indicated under each physiological variable. * indicates that logarithms of data have been used for the analysis. n.s., not significant.

**Table 4:** Regression analysis for the physiological variables studied and those genera with statistical differences in total percentage.

Genera	FI 0.34/0.0299	WI 0.64/0.0001	Diuresis 0.40/0.0027	BW 0.13/0.0414	SBP 0.21/0.0093	Leptin 0.45/0.0002	Insulin * 0.14/0.0296	Triglycerides * 0.14/0.0323	T-CHO 0.39/0.0018	HDL/LDL* 0.50/0.0009
Desulfovibrio	1.81 ± 0.57 (0.0034)	4.49 ± 1.35 (0.0028)	1.36 ± 0.46 (0.0075)	n.s.	n.s.	n.s.	n.s.	n.s.	48.34 ± 13.62 (0.0013)	n.s.
Ruminiclostridium	n.s.	−1.41 ± 0.48 (0.0074)	n.s.	n.s.	8.43 ± 3.02 (0.0093)	n.s.	n.s.	n.s.	n.s.	0.13 ± 0.06 (0.0295)
Fusicatenibacter	n.s.	13.80 ± 4.05 (0.0023)	4.78 ± 1.63 (0.0074)	−6.34 ± 2.98 (0.0414)	n.s.	n.s.	n.s.	n.s.	−142.93 ± 37.67 (0.0007)	−1.84 ± 0.45 (0.0004)
Parasutterella	n.s.	n.s.	n.s.	n.s.	n.s.	n.s.	−0.94 ± 0.41 (0.0296)	n.s.	n.s.	−1.01 ± 0.29 (0.0020)
Olivibacter	−6.32 ± 2.78 (0.0310)	n.s.	n.s.	n.s.	n.s.	n.s.	n.s.	2.80 ± 1.25 (0.0324)	n.s.	n.s.
Marispirillum	n.s.	n.s.	n.s.	n.s.	n.s.	−3.21 ± 0.89 (0.0012)	n.s.	n.s.	n.s.	n.s.
Spiroplasma	−2.94 ± 1.18 (0.0189)	n.s.	n.s.	n.s.	n.s.	n.s.	n.s.	n.s.	n.s.	−0.85 ± 0.39 (0.0394)
Marinilabilia	16.90 ± 7.08 (0.0239)	90.74 ± 17.52 (0.0000)	18.61 ± 8.01 (0.0293)	n.s.	n.s.	n.s.	n.s.	n.s.	n.s.	n.s.
Desulfotomaculum	−34.26 ± 14.03 (0.0212)	n.s.	n.s.	n.s.	n.s.	n.s.	n.s.	n.s.	−764.08 ± 346.92 (0.0355)	n.s.
Helicobacter	n.s.	200.22 ± 68.19 (0.0072)	81.99 ± 27.92 (0.0074)	n.s.	n.s.	−28.18 ± 12.44 (0.0314)	n.s.	n.s.	n.s.	n.s.

For each case, regression coefficient estimate, s.e. and p values are shown. *R*^2^ and p values of the model are also indicated under each physiological variable. * indicates that logarithms of data have been used for the analysis. FI, food intake; WI, water intake; T-CHO, total cholesterol; SBP, systolic blood pressure. n.s., not significant.

## Supplementary Materials

The following are available online at https://www.mdpi.com/2076-2607/7/2/61/s1, Table S1: *p*-values of Kruskal–Wallis test, and pairwise comparisons of nonadjusted and adjusted *p*-values of the families with significant differences between diets in the percentage of sequences retrieved from fecal samples.

## References

[1] Riccardi G., Giacco R., Rivellese A.A. (2004). Dietary fat, insulin sensitivity and the metabolic syndrome. Clin. Nutr..

[2] Arumugam M., Raes J., Pelletier E., Le Paslier D., Yamada T., Mende D.R., Fernandes G.R., Tap J., Bruls T., Batto J.M. (2011). Enterotypes of the human gut microbiome. Nature.

[3] Albenberg L.G., Wu G.D. (2014). Diet and the intestinal microbiome: Associations, functions, and implications for health and disease. Gastroenterology.

[4] Zhang C., Zhang M., Wang S., Han R., Cao Y., Hua W. (2010). Interactions between gut microbiota, host genetics and diet relevant to development of metabolic syndromes in mice. ISME J..

[5] Sunkara T., Rawla P., Ofosu A., Gaduputi V. (2018). Fecal microbiota transplant—A new frontier in inflammatory bowel disease. J. Inflamm. Res..

[6] Poudyal H., Panchal S.K., Diwan V., Brown L. (2011). Omega-3 fatty acids and metabolic syndrome: Effects and emerging mechanisms of action. Prog. Lipid Res..

[7] Huang C.W., Chien Y.S., Chen Y.J., Ajuwon K.M., Mersmann H.M., Ding S.T. (2016). Role of n-3 Polyunsaturated Fatty Acids in Ameliorating the Obesity-Induced Metabolic Syndrome in Animal Models and Humans. Int. J. Mol. Sci..

[8] Fava F., Gitau R., Griffin B.A., Gibson G.R., Tuohy K.M., Lovegrove J.A. (2013). The type and quantity of dietary fat and carbohydrate alter faecal microbiome and short-chain fatty acid excretion in a metabolic syndrome ‘at-risk’ population. Int. J. Obes..

[9] Gillingham L.G., Harris-Janz S., Jones P.J. (2011). Dietary monounsaturated fatty acids are protective against metabolic syndrome and cardiovascular disease risk factors. Lipids.

[10] Martínez-González M.A., Martín-Calvo N. (2013). The major European dietary patterns and metabolic syndrome. Rev. Endocr. Metab. Disord..

[11] Hidalgo M., Prieto I., Abriouel H., Cobo A., Benomar N., Gálvez A., Martínez-Cañamero M. (2014). Effect of virgin and refined olive oil consumption on gut microbiota. Comparison to butter. Food Res. Int..

[12] Prieto I., Hidalgo M., Segarra A.B., Martínez-Rodríguez A.M., Cobo A., Ramírez M., Abriouel H., Gálvez A., Martínez-Cañamero M. (2018). Influence of a diet enriched in virgin olive oil or butter on mouse gut microbiota and its correlation to physiological and biochemical parameters related to metabolic syndrome. PLoS ONE.

[13] Medina E., de Castro A., Romero C., Brenes M. (2006). Comparison of the concentrations of phenolic compounds in olive oils and other plant oils: Correlation with antimicrobial activity. J. Agric. Food Chem..

[14] Grossi C., Rigacci S., Ambrosini S., Dami T.E., Luccarini I., Traini C., Failli P., Berti A., Casamenti F., Stefani M. (2013). The polyphenol oleuropein aglycone protects TgCRND8 mice against Aß plaque pathology. PLoS ONE.

[15] Anhê F.F., Roy D., Pilon G., Dudonné S., Matamoros S., Varin T.V., Garofalo C., Moine Q., Desjardins Y., Levy E. (2015). A polyphenol-rich cranberry extract protects from diet-induced obesity, insulin resistance and intestinal inflammation in association with increased Akkermansia spp. population in the gut microbiota of mice. Gut.

[16] Roopchand D.E., Carmody R.N., Kuhn P., Moskal K., Rojas-Silva P., Turnbaugh P.J., Raskin I. (2015). Dietary Polyphenols Promote Growth of the Gut Bacterium Akkermansia muciniphila and Attenuate High-Fat Diet-Induced Metabolic Syndrome. Diabetes.

[17] Ramírez-Tortosa C., López-Pedrosa J.M., Suarez A., Ros E., Mataix J., Gil A. (1999). Olive oil- and fish oil-enriched diets modify plasma lipids and susceptibility of LDL to oxidative modification in free-living male patients with peripheral vascular disease: The Spanish Nutrition Study. Br. J. Nutr..

[18] Gorzynik-Debicka M., Przychodzen P., Cappello F., Kuban-Jankowska A., Marino Gammazza A., Knap N., Wozniak M., Gorska-Ponikowska M. (2018). Potential health benefits of olive oil and plant polyphenols. Int. J. Mol. Sci..

[19] Ministerio de Agricultura y Pesca, Alimentación y Medio Ambiente (2017). Informe del Consumo de Alimentación en España 2016.

[20] Vázquez-Araújo L., Adhikari K., Chambers E., Chambers D.H., Carbonell-Barrachina A.A. (2015). Cross-cultural perception of six commercial olive oils: A study with Spanish and US consumers. Food Sci. Technol. Int..

[21] Pacheco Y.M., López S., Bermúdez B., Abia R., Muriana F.J. (2006). Extra-virgin vs. refined olive oil on postprandial hemostatic markers in healthy subjects. J. Thromb. Haemost..

[22] Banegas I., Prieto I., Vives F., Alba F., de Gasparo M., Duran R., de Dios Luna J., Segarra A.B., Hermoso F., Ramírez M. (2009). Asymmetrical response of aminopeptidase A and nitric oxide in plasma of normotensive and hypertensive rats with experimental hemiparkinsonism. Neuropharmacology.

[23] Segarra A.B., Ramirez M., Banegas I., Alba F., Vives F., de Gasparo M., Ortega E., Ruiz E., Prieto I. (2008). Dietary fat influences testosterone, cholesterol, aminopeptidase A, and blood pressure in male rats. Horm. Metab. Res..

[24] Liu M.Y., Xydakis A.M., Hoogeveen R.C., Jones P.H., Smith E.B., Nelson K.W., Ballantyne C.M. (2005). Multiplexed analysis of biomarkers related to obesity and the metabolic syndrome in human plasma, using the Luminex-100 system. Clin. Chem..

[25] Nocella C., Cammisotto V., Fianchini L., D’Amico A., Novo M., Castellani V., Stefanini L., Violi F., Carnevale R. (2018). Extra virgin olive oil and cardiovascular diseases: Benefits for human health. Endocr. Metab. Immune Disord. Drug Targets.

[26] Hidalgo M., Prieto I., Abrioue H., Villarejo A.B., Ramírez M., Cobo A., Benomar N., Gálvez A., Martínez-Cañamero M. (2018). Changes in Gut Microbiota Linked to a Reduction in Systolic Blood Pressure in Spontaneously Hypertensive Rats Fed an Extra Virgin Olive Oil-Enriched Diet. Plant Foods Hum. Nutr..

[27] Faith J.J., McNulty N.P., Rey F.E., Gordon J.L. (2011). Predicting a human gut microbiota’s response to diet in gnotobiotic mice. Science.

[28] Rey F.E., Gonzalez M.D., Cheng J., Wu M., Ahern P.P., Gordon J.I. (2013). Metabolic niche of a prominent sulfate-reducing human gut bacterium. Proc. Natl. Acad. Sci. USA.

[29] Devkota S., Chang E.B. (2015). Interactions between Diet, Bile Acid Metabolism, Gut Microbiota, and Inflammatory Bowel Diseases. J. Dig. Dis..

[30] Freeman B.A., Sissenstein R., McManus T.T., Woodward J.E., Lee I.M., Mudd J.B. (1976). Lipid composition and lipid metabolism of Spiroplasma citri. J. Bacteriol..

[31] Shirley H.Y., Hung T.A., Chen R.F., Whitcomb J.G., Tully Y.X. (1987). *Spiroplasma culicicola* sp. nov. from the Salt Marsh Mosquito Aedes sollicitanst. Int. J. Syst. Evol. Microbiol..

[32] Musetti R., Favali M.A., Pressacco L. (2000). Histopathology and polyphenol content in plants infected by phytoplasmas. Cytobios.

[33] Furneri P.M., Piperno A., Sajia A., Bisignano G. (2004). Antimycoplasmal activity of hydroxytyrosol. Anticancer Agents Med. Chem..

[34] Shen Z., Xu S., Dewhirst F.E., Paster B.J., Pena J.A., Modlin I.M., Kidd M., Fox J.G. (2005). A novel enterohepatic Helicobacter species ‘Helicobacter mastomyrinus’ isolated from the liver and intestine of rodents. Helicobacter.

[35] Hildebrandt M.A., Hoffmann C., Sherrill–Mix S.A., Keilbaugh S.A., Hamady M., Chen Y.Y., Knight R., Ahima R.S., Bushman F., Wu G.D. (2009). High-fat diet determines the composition of the murine gut microbiome independently of obesity. Gastroenterology.

[36] Cani P.D., Amar J., Iglesias M.A., Poggi M., Knauf C., Bastelica D. (2007). Metabolic endotoxemia initiates obesity and insulin resistance. Diabetes.

