# Supplementary material for: Refined versus Extra Virgin Olive Oil High-Fat Diet Impact on Intestinal Microbiota of Mice and Its Relation to Different Physiological Variables
Source: Microorganisms. 2019 Feb 23;7(2):61. doi: 10.3390/microorganisms7020061 (PMC6406240; doi:10.3390/microorganisms7020061)
Supplement: Supplementary file 1 [file microorganisms-07-00061-s001.pdf]

Table S1. p-values of Kruskal-Wallis test, and pairwise comparisons non-adjusted and adjusted *p*-values of the families with significant differences between diets in the percentage of sequences retrieved from faecal samples.

Family	Significance	Non-Adjusted Pairwise Significance Level	Adjusted Pairwise Significance Level
Prevotellaceae	0.027	EVOO vs BT 0.370	EVOO vs BT 1.000
		EVOO vs SD 0.003	**EVOO vs SD 0.019**
		EVOO vs ROO 0.334	EVOO vs ROO 1.000
		ROO vs BT 0.945	ROO vs BT 1.000
		ROO vs SD 0.045	ROO vs SD 0.268
		BT vs SD 0.038	BT vs SD 0.228
Desulfovibrionaceae	0.023	EVOO vs BT 0.020	EVOO vs BT 0.117
		EVOO vs SD 0.686	EVOO vs SD 1.000
		EVOO vs ROO 0.015	EVOO vs ROO 0.089
		ROO vs BT 0.918	ROO vs BT 1.000
		ROO vs SD 0.050	ROO vs SD 0.299
		BT vs SD 0.063	BT vs SD 0.377
Marinilabiliaceae	0.003	EVOO vs BT 0.612	EVOO vs BT 1.000
		EVOO vs SD 0.018	EVOO vs SD 0.109
		EVOO vs ROO 0.245	EVOO vs ROO 1.000
		ROO vs BT 0.511	ROO vs BT 1.000
		ROO vs SD 0.000	**ROO vs SD 0.003**
		BT vs SD 0.004	**BT vs SD 0.026**
Erysipelotrichaceae	0.013	EVOO vs BT 0.038	EVOO vs BT 0.231
		EVOO vs SD 0.002	**EVOO vs SD 0.009**
		EVOO vs ROO 0.023	EVOO vs ROO 0.137
		ROO vs BT 0.836	ROO vs BT 1.000
		ROO vs SD 0.340	ROO vs SD 1.000
		BT vs SD 0.248	BT vs SD 1.000
Sutterellaceae	0.041	EVOO vs BT 0.290	EVOO vs BT 1.000
		EVOO vs SD 0.006	**EVOO vs SD 0.037**
		EVOO vs ROO 0.059	EVOO vs ROO 0.355
		ROO vs BT 0.407	ROO vs BT 1.000
		ROO vs SD 0.362	ROO vs SD 1.000
		BT vs SD 0.086	BT vs SD 0.517
Christensenellaceae	0.026	EVOO vs BT 0.012	EVOO vs BT 0.071
		EVOO vs SD 0.007	**EVOO vs SD 0.040**
		EVOO vs ROO 0.128	EVOO vs ROO 0.766
		ROO vs BT 0.321	ROO vs BT 1.000
		ROO vs SD 0.218	ROO vs SD 1.000
		BT vs SD 0.788	BT vs SD 1.000
Spiroplasmataceae	0.013	EVOO vs BT 0.685	EVOO vs BT 1.000
		EVOO vs SD 0.346	EVOO vs SD 1.000
		EVOO vs ROO 0.021	EVOO vs ROO 0.128
		ROO vs BT 0.058	ROO vs BT 0.346
		ROO vs SD 0.001	**ROO vs SD 0.009**
		BT vs SD 0.182	BT vs SD 1.000

Staphylococcaceae	0.013	EVOO vs BT 0.223<br>EVOO vs SD 0.126<br>EVOO vs ROO 0.114<br>ROO vs BT 0.005<br>ROO vs SD 1.000<br>BT vs SD 0.007	EVOO vs BT 1.000<br>EVOO vs SD 0.753<br>EVOO vs ROO 0.686<br>**ROO vs BT 0.031**<br>ROO vs SD 1.000<br>**BT vs SD 0.040**
Microbacteriaceae	0.001	EVOO vs BT 0.001<br>EVOO vs SD 1.000<br>EVOO vs ROO 1.000<br>ROO vs BT 0.001<br>ROO vs SD 1.000<br>BT vs SD 0.001	**EVOO vs BT 0.006**<br>EVOO vs SD 1.000<br>EVOO vs ROO 1.000<br>**ROO vs BT 0.006**<br>ROO vs SD 1.000<br>**BT vs SD 0.008**
Helicobacteriaceae	0.034	EVOO vs BT 0.461<br>EVOO vs SD 0.475<br>EVOO vs ROO 0.067<br>ROO vs BT 0.010<br>ROO vs SD 0.013<br>BT vs SD 1.000	EVOO vs BT 1.000<br>EVOO vs SD 1.000<br>EVOO vs ROO 0.401<br>ROO vs BT 0.061<br>ROO vs SD 0.076<br>BT vs SD 1.000
